# Exploring the Neuropsychiatric Sequalae of Perceived COVID-19 Exposure in College Students: A Pilot Digital Phenotyping Study

**DOI:** 10.3389/fpsyt.2021.788926

**Published:** 2022-01-10

**Authors:** Suraj K. Patel, John Torous

**Affiliations:** The Division of Digital Psychiatry, Beth Israel Deaconess Medical Center, Harvard Medical School, Boston, MA, United States

**Keywords:** COVID-19, smartphone application, psychosis, mood, students

## Abstract

The urgency to understand the long-term neuropsychiatric sequala of COVID-19, a part of the Post-Acute COVID-19 Syndrome (PACS), is expanding as millions of infected individuals experience new unexplained symptoms related to mood, anxiety, insomnia, headache, pain, and more. Much research on PACS involves cross sectional surveys which limits ability to understand the dynamic trajectory of this emerging phenomenon. In this secondary analysis, we analyzed data from a 4-week observational digital phenotyping study using the mindLAMP app for 695 college students with elevated stress who specified if they were exposed to COVID-19. Students also completed a biweekly survey of clinical assessments to obtain active data. Additionally, passive data streams like GPS, accelerometer, and screen state were extracted from phone sensors and through features the group built. Three hundred and eighty-second number participants successfully specified their COVID-19 exposure and completed the biweekly survey. From active smartphone data, we found significantly higher scores for the Prodromal Questionnaire (PQ) and the Pittsburgh Sleep Quality Index (PSQI) for students reporting exposure to COVID-19 compared to those who were not (*p*s < 0.05). Additionally, we found significantly decreased sleep duration as captured from the smartphone via passive data for the COVID-19 exposed group (*p* < 0.05). No significant differences were detected for other surveys or passive sensors. Smartphones can capture both self-reported symptoms and behavioral changes related to PACS. Our results around changes in sleep highlight how digital phenotyping methods can be used in a scalable and accessible manner toward better capturing the evolving phenomena of PACS. The present study further provides a foundation for future research to implement improving digital phenotyping methods.

## Introduction

As the neuropsychiatric sequalae of COVID-19 become clearer, the urgency to both better understand this cluster of symptoms and respond with scalable interventions expands. An April 2021 study from Lancet Psychiatry estimated a 33% incidence for neurological or psychiatric diagnosis in the following 6-months after COVID-19 exposure (1). Symptoms from Post-Acute COVID-19 Syndrome (PACS), popularly known as “long COVID,” include headache, anxiety, fatigue, and mood symptoms in addition to anosmia, insomnia, myalgia, dysgeusia, and weakness (2). New longitudinal research suggests symptoms vary in both severity and prevalence over time with fatigue and cognitive dysfunction as the most prominent symptoms (3). These diverse symptoms and their temporal dynamics thus require multimodal and longitudinal monitoring for which monitoring via smartphone mobile apps is well-suited.

Advantages of smartphone monitoring via digital phenotyping, the “moment-by-moment quantification of the individual-level human phenotype *in situ* using data from personal digital devices” for understanding PACS are highlighted when compared to cross sectional methods that are often employed instead today. Cross sectional methods are only able to detect self-reported symptoms at a certain time point and subject to retrospective recall which is especially of note given cognitive impairment is a common symptom of PACS. Given this is likely a longitudinal syndrome that is dynamic and changes with time and perhaps environment, it is thus not surprising that cross sectional reports are often conflicting. For example, some studies have found higher rates of depression (4) and anxiety (5) during the pandemic while some other have conflicting reports of sometimes even lower rates of depression (6) and anxiety (7, 8) when comparing symptom rates from before the pandemic. Thus, cross-sectional data only paints a partial picture of how COVID-19 may affect symptomology.

Digital phenotyping methods instead allow data capture of repeated surveys but also continuous collection of sensors that be help inform behavioral, cognitive, and physiological symptoms as well. In prior research our team has shown how we can transform smartphone geolocation data into metrics related to home time, mobility patterns, and entropy; accelerometer into metrics related to sleep, sedentary, and activity periods; screen taps into metrics of memory and attention, and screen on/off logs into metrics of screen time. This multimodal data offers clear potential for understanding the evolving symptoms of PACS. Further, digital phenotyping methods allow to close gaps created by partial understanding of PACS from cross-sectional studies.

Among studying how COVID-19 affects various populations, young people are of important interest. Younger people may be at greater risks of the neuropsychiatric effects of COVID-19 because of how disruptive the pandemic has been to previously stable lifestyles, which could lead to greater rates of anxiety and depression (9). College students have also been found to generally display greater rates of anxiety, depression, and trouble sleeping (4). These mental health troubles may also contribute to decreased academic performance based on findings of associations between emotional self-regulation and greater academic performance during COVID-19 (10). However, as younger populations often also have the highest rates of smartphone ownership, they have a greater interest in using technology to better understand and improve their health.

In this study we aimed to explore if there are differences in psychiatric symptoms based on COVID-19 exposure in students using smartphones to capture two types of longitudinal data: active and passive. Passive data includes home time, sleep duration, and screen duration and is useful to study as decreased sleep, fatigue, and myalgia are known symptoms of PACS (3, 11). In line with clinical reports and a passive data study using a wearable to estimate sleep (12), we hypothesize those exposed would sleep less on average and that we could detect this change now from smartphone passive data. As an exploratory hypothesis, we propose that people exposed to COVID-19 may have spent less time at home. As a control, we propose that total screen duration should not be associated with COVID-19 exposure status as there is no clear literature that use of technology is any greater or less based on exposure.

Overall, the objective of this study is to examine how traditional survey and novel digital phenotyping methods can be used to understand the difference in neuropsychiatric symptoms based on perceived COVID-19 exposure. Literature has shown how symptoms associated with PACS may be related to COVID-19 exposure, thus this study will further highlight how current and future studies can implement similar methods and expand upon usage of current digital phenotyping techniques. This will allow for practitioners and researchers alike to be exposed to more comprehensive approaches for implementing and understanding contemporary mental health care.

## Methods

The present study is a secondary analysis of a digital phenotyping of college students, which used the opensource mindLAMP app. The app is under regular development and maintains quality and stability checks for both iPhone and Android versions on a bi-weekly basis. From November 2020 to May 2021, 695 college students were recruited for a 4-week study through university Reddit forums to use the mindLAMP app to take daily and bi-weekly surveys about their mental health for $50 over the study. Six hundred and twelfth number students successfully went on to use the app. Recruitment was through online advertisements for a paid mental health study for college students. Recruited students then took an enrollment survey through REDCap of the Perceived Stress Scale (PSS) (13). Additionally, the enrollment survey included a demographic questionnaire with the following question to specify if they were exposed to or unsure of COVID-19 exposure: “*Have you had COVID-19?”*, to which participants could respond “Yes,” “No,” and “Unsure, but I think so.” Eligibility criteria consisted of (1) fluency in English, (2) ownership either of a capable iPhone or Android smartphone to run the study app, (3) college student verification with a college email and student ID card, and (4) a PSS score of 14 or higher. PSS reports of 14 or higher indicate at least moderate stress. The PSS threshold was used to screen for students who were already at elevated levels of stress to exclude healthy students which could skew results of average reports for stressed students. Of the 612 participants, 507 completed their weekly surveys. Of the 507 participants with survey data, 382 had responded to the COVID-19 exposure question and were included for the present study's analysis.

### Measures

#### Active Data/Surveys

Enrolled students received a daily survey of 11 items with questions from various gold standard clinical assessments to gauge their mental health on an everyday basis. The assessments drawn from are the Patient Health Questionnaire (PHQ-9), the General Anxiety Disorder 7 questionnaire (GAD-7), the Prodromal Questionnaire (PQ), and the PSS. Participants also received a longer bi-weekly survey which included full-length assessments for the PHQ-9, the GAD-7, the PSS, the UCLA Loneliness scale, the PQ, the Pittsburgh Sleep Quality Index (PSQI), and the Digital Working Alliance Inventory (D-WAI). The bi-weekly survey data was used for analyses.

The selected surveys were chosen for various relevant reasons. The PHQ-9 and GAD-7 are often used as assessments for mood and anxiety, respectively, which as often cited as elevated in COVID-19 patients and during the overall pandemic. The PQ is used to detect early onset psychotic symptoms, as psychotic symptoms have been seen manifesting in COVID-19-related conditions (14). The PSQI is used a self-report sleep measure that is often cited in COVID-19 symptoms (15). The UCLA Loneliness scale measures reports of loneliness that could result from social isolation faced during quarantine (4). Lastly, the D-WAI was used as a quality metric to assess participant interactions with the mindLAMP app (16).

#### Passive Data/Sensors

Passive sensor data was also collected for the duration of the study. Accepted permissions for phone sensors like GPS, screen duration, and motion are referred to as raw features. This passive data was then used to create various markers for analysis through mindLAMP's regularly updated data analysis pipeline, Cortex which is freely available at https://github.com/BIDMCDigitalPsychiatry/LAMP-cortex. Primary features, for example, include “Significant Locations,” which processes raw GPS data and groups data points together into weighted traveled regions of significance. This data was then further developed into secondary features like home time, which buckets Significant Locations by the specified resolution and determines the amount of time an individual spent at home within that time window. The present study looks at the passive features of home time, sleep duration, and screen duration. Sleep duration was a feature created by making an optimal sleep window by finding the 8-h period within the overnight interval [18:00, 10:00] which minimized accelerometer activity across all nights of their participation. To estimate nightly sleep durations, the optimal window was adjusted based on the accelerometer activity for the specific night. Screen duration was computed using accelerometer/motion and screen state data from the inherent smartphone metric by summing the bouts of time where the phone went from being in the “on” to the “off” state.

#### Analysis

All statistical analyses and visualizations were carried out using the R (version 4.1.1) packages “*psych*” (17), and “*ggplot2*” (18). Participants were split into groups based on COVID-19 exposure from the screening survey, with one group consisting of students who were not exposed to COVID-19 and another group for students who were either exposed to COVID-19 or were unsure if they were exposed or not. These two groups had their biweekly survey scores compared from an individual survey level. Averages for the entries each participant completed were taken from the biweekly surveys and then stratified based on each individual survey the biweekly survey was composed of. Survey averages were normalized to be put on the same scale since individual surveys differed in length. A series of two-sample *t*-tests were carried out to compare individual surveys between COVID-19 exposure groups. For passive data, sensor means based on COVID-19 exposure were compared.

## Results

**Figure 1:**
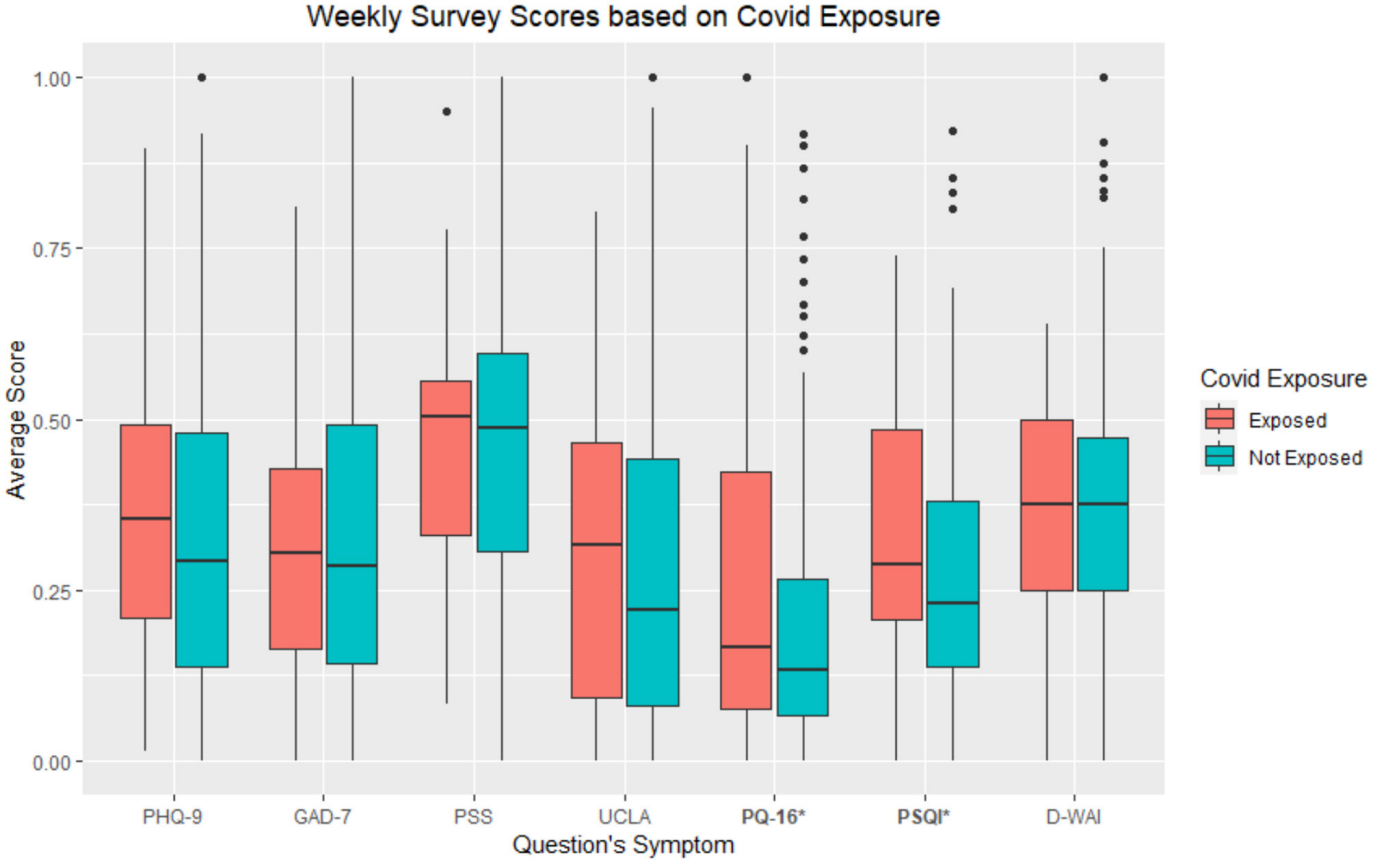
Average individual survey scores from larger biweekly survey based on COVID-19 exposure groups. As the app offered daily surveys similar to the larger assessments, “_W” at the end of each survey indicates the survey being a part of the biweekly survey. *refers to *p* < 0.05.

Of the 382 participants, 237 (62%) identified as female and 133 (35%) identified as male, with 11 (3%) reporting identification as a different gender identity After testing for differences in each individual survey based on perceived COVID-19 exposure, the exposed group (M = 11.82, SD = 11.53) compared to the non-exposed groups (M = 8.67, SD = 8.69) had significantly higher PQ-16 scores, *t*_(380)_ = 2.34, *p* < 0.05. In addition, it was found that the exposed group (M = 7.13, SD = 4.26) compared to the non-exposed groups (M = 5.84, SD = 3.82) had significantly higher PSQI scores, *t*_(380)_ = 2.27, *p* < 0.05. See Figure 1 for visual comparison of all surveys between exposure groups. Significantly different survey scores are starred.

**Figure 2:**
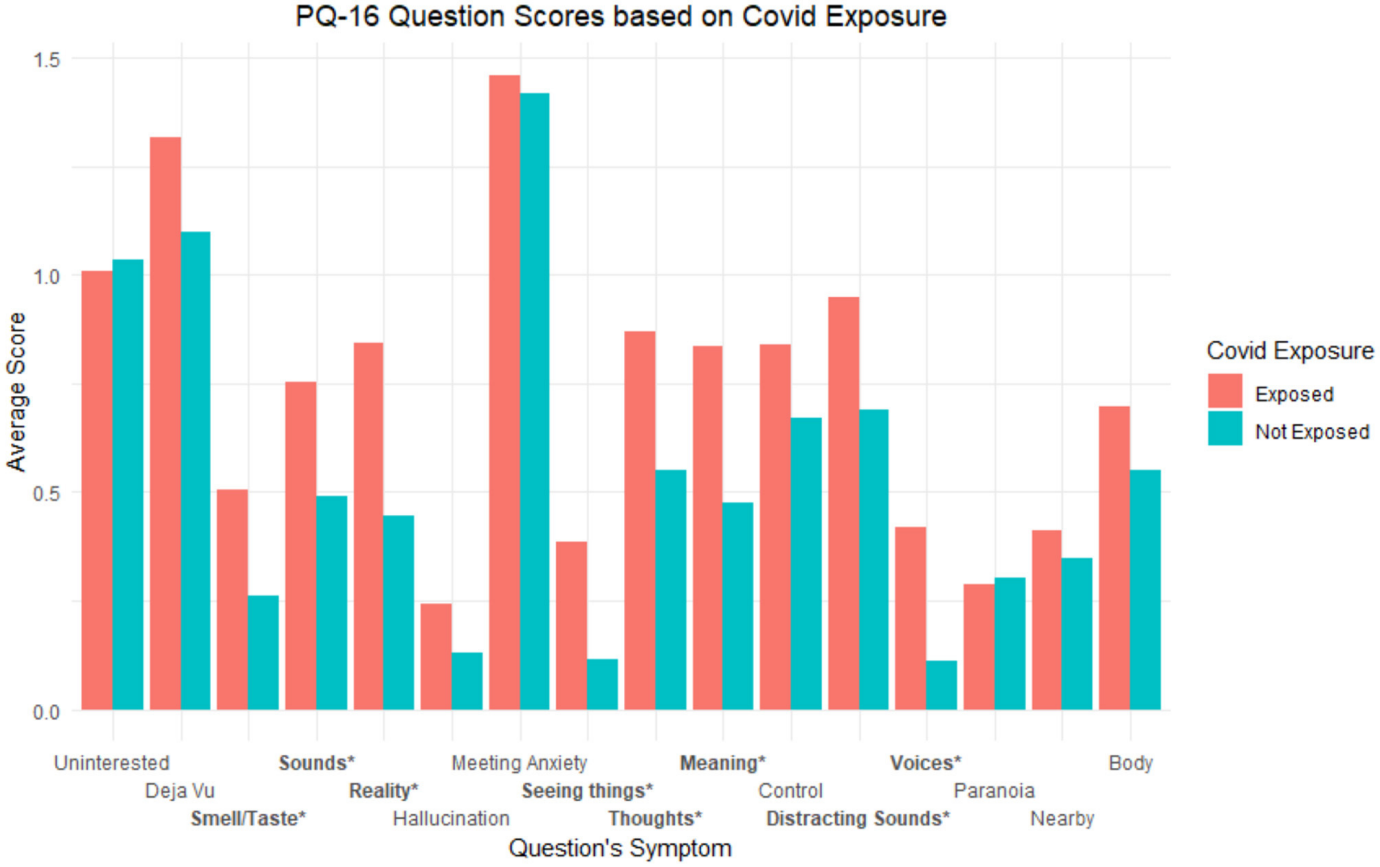
Average PQ-16 question scores based on exposure groups. Labels are coded based on the symptom being assessed. * refers to *p* < 0.05 and ** refers to *p* < 0.001.

After further question level breakdown, we found significantly greater scores based on perceived COVID-19 exposure in the PQ-16 for the following items coded based on the symptom being assessed (*p*s < 0.05): “Smell/Taste,” “Sounds,” “Reality,” “Seeing things,” “Thoughts,” “Meaning,” “Distracting Sounds.” See Figure 2 for a graphical depiction of average question scores by exposure group with significant symptoms starred.

**Figure 3:**
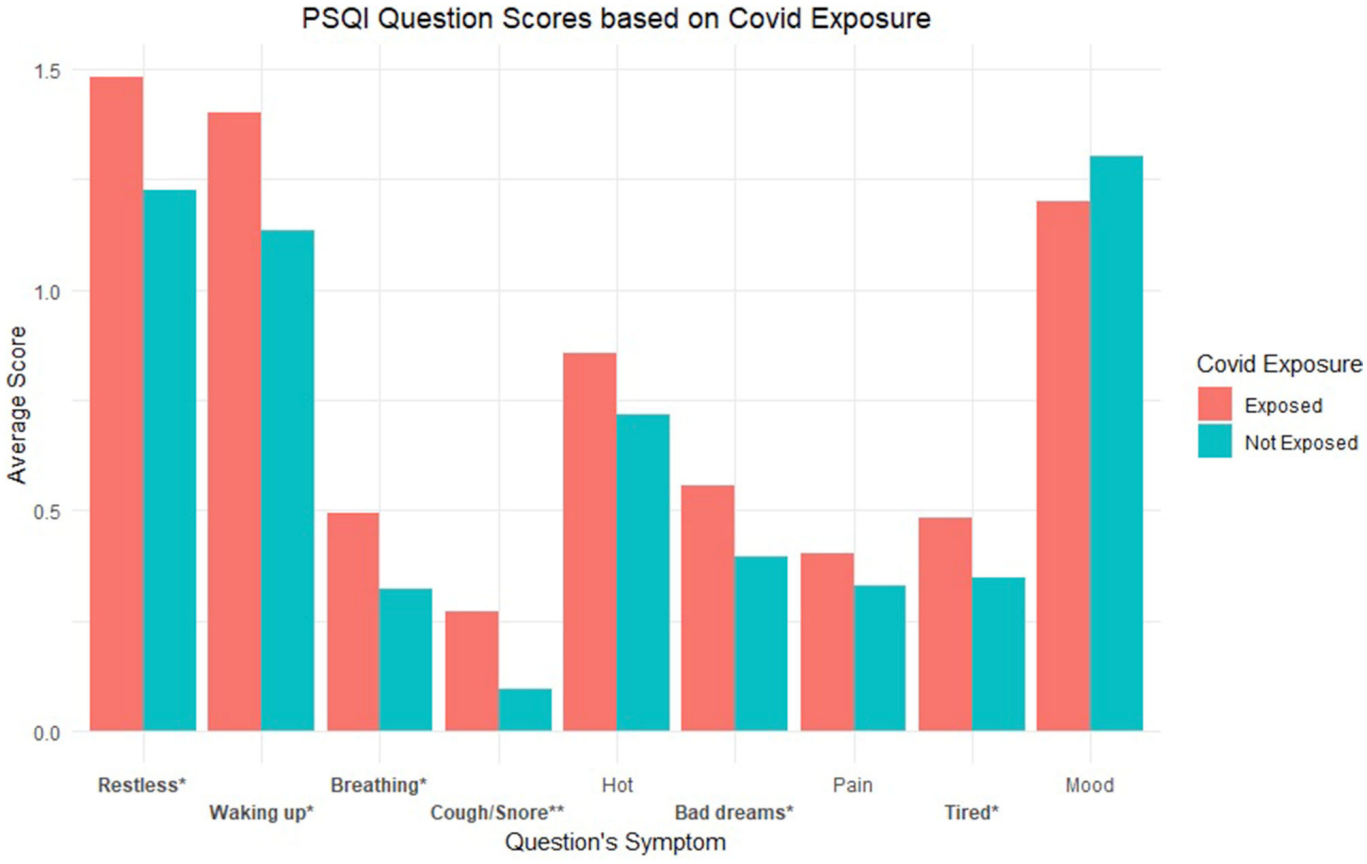
Average PSQI question scores based on exposure groups. Labels are coded based on the symptom being assessed. * refers to *p* < 0.05 and ** refers to *p* < 0.001.

Question level breakdown for the PSQI showed significant differences based on perceived COVID-19 exposure for the following items coded based on the symptoms the question assessed (*p*s < 0.05): “Restless,” “Waking up,” “Breathing,” “Cough/Snore,” “Bad dreams,” “Tired.” See Figure 3 for a graphical depiction of average question scores by exposure group with significant symptoms starred. Additionally, see Table 1 for all *t*-test statistics for both PQ-16 and PSQI related analyses.

**Table 1:** Comprehensive results for all t-tests for individual question breakdowns for PQ16 and PSQI.

	**Code**	**Question**	**COVID-19 exposed**	**COVID-19 not exposed**	***t***	***P***	**95% CI**	
							**Lower bound**	**Upper bound**
PQ16	Uninterested	*I feel uninterested in the things I used to enjoy*	1.01 (1.29)	1.03 (1.26)	−0.14	0.56	−0.33	0.28
	Deja Vu	*I often seem to live through events exactly as they happened before*.	1.31 (1.32)	1.09 (1.26)	1.17	0.12	−0.09	0.53
	Smell/Taste	*I sometimes smell or taste things that other people can't smell or taste*.	0.50 (0.99)	0.26 (0.70)	2.22	<0.05^*^	0.06	0.43
	Sounds	*I often hear unusual sounds like banging, clicking, hissing, clapping or ringing in my ears*.	0.75 (1.16)	0.49 (1.01)	1.73	<0.05^*^	0.01	0.51
	Reality	*I have been confused at times whether something I experienced was real or imaginary*.	0.42 (0.97)	0.11 (0.47)	3.68	<0.001^**^	0.16	0.64
	Hallucination	*When I look at a person, or look at myself in a mirror, I have seen the face change right before my eyes*.	0.24 (0.80)	0.13 (0.52)	1.39	0.08	−0.02	0.25
	Meeting anxiety	*I get extremely anxious when meeting people for the first time*.	1.46 (1.35)	1.39 (1.42)	0.21	0.42	−0.29	0.38
	Seeing things	*I have seen things that other people apparently can't see*.	0.38 (0.90)	0.12 (0.51)	3.14	<0.001^**^	0.13	0.41
	Thoughts	*My thoughts are sometimes so strong that I can almost hear them*.	0.87 (1.26)	0.55 (1.01)	2.06	<0.05^*^	0.06	0.57
	Meaning	*I sometimes see special meanings in advertisements, shop windows, or in the way things are arranged around me*.	0.84 (1.21)	0.48 (0.95)	2.47	<0.05^*^	0.12	0.6
	Control	*Sometimes I have felt that I'm not in control of my own ideas or thoughts*.	0.84 (1.17)	0.67 (1.11)	1.04	0.15	−0.09	0.44
	Distracting sounds	*Sometimes I feel suddenly distracted by distant sounds that I am not normally aware of*	0.95 (1.26)	0.69 (1.11)	1.55	0.06	−0.02	0.53
	Voices	*I have heard things other people can't hear like voices of people whispering or talking*.	0.84 (1.21)	0.44 (0.93)	2.79	<0.05^*^	0.17	0.45
	Paranoia	*I often feel that others have it in for me*.	0.29 (0.76)	0.30 (0.79)	−0.13	0.55	−0.2	0.18
	Nearby	*I have had the sense that some person or force is around me, even though I could not see anyone*.	0.41 (0.85)	0.35 (0.86)	0.52	0.3	−0.14	0.27
	Body	*I feel that parts of my body have changed in some way, or that parts of my body are working differently than before*.	0.69 (1.11)	0.55 (1.00)	0.99	0.16	−0.1	0.39
PSQI	Restless	*How often is it that you cannot get to sleep within 30 min*	1.48 (1.08)	1,22 (0.99)	1.74	<0.05^*^	0.01	0.49
	Waking up	*How often is it that you wake up in the middle of the night or early morning*,	1.40 (0.99)	1.13 (0.96)	1.88	<0.05^*^	0.33	0.5
	Breathing	*How often have you had trouble sleeping because you cannot breathe comfortably*	0.49 (0.75)	0.32 (0.62)	1.85	<0.05^*^	0.02	0.33
	Cough/Snore	*How often have you had trouble sleeping because you cough or snore loudly*	0.27 (0.58)	0.09 (0.26)	2.98	<0.001^*^	0.08	0.27
	Hot	*How often have you had trouble sleeping because you feel too hot*	0.86 (0.77)	0.72 (0.73)	1.29	0.09	−0.04	0.32
	Bad dreams	*How often have you had trouble sleeping because you have bad dreams*	0.55 (0.71)	0.39 (0.63)	1.73	<0.05^*^	<0.01	0.32
	Pain	*How often have you had trouble sleeping because you have pain*	0.40 (0.57)	0.33 (0.59)	0.84	0.19	−0.07	0.22
	Tired	*During the past week, how often have you had trouble staying awake while driving, eating meals, or engaging in social activity?*	0.48 (0.59)	0.35 (0.56)	1.65	<0.05^*^	<0.01	0.27
	Mood	*During the past week, how much of a problem has it been for you to keep up enthusiasm to get things done?*	1.19 (0.85)	1.30 (0.92)	−0.77	0.78	−0.32	0.12

*Formatting for Group Statistics are Mean (Standard Deviation).*

* *Indicates p < 0.05,*

** *indicates p < 0.001*.

For passive data, average sleep duration differed between groups based on COVID-19 exposure, with students who had been or were unsure of their exposure had a lower average sleep duration in hours (M = 6.77, SD = 1.54) than students who were not exposed (M = 7.25, SD = 1.60), *t*_(425)_ = −2.11, *p* < 0.05 Differences in home time and screen duration were not significantly different across exposure groups (*p* > 0.05).

## Discussion

Using digital phenotyping methods to collect longitudinal data in 382 college students for ~30 days, we showed significant differences in passive data sleep duration and self-reported sleep and psychosis assessments among self-reported COVID-19 exposed students as compared to unexposed. These results around sleep and psychosis also match ongoing research and suggest how passive data may complement clinical characterization and offer potentially useful data toward understanding symptoms and functional outcomes related to illness.

The combination of active and passive sensor capture smartphone apps may help elucidate how COVID-19 exposure affects both mental and physical health. For example, our results around both subjective and more objective measures related to sleep offer the ability to examine different facets of people's experience of the illness. While correlational, the finding that those with self-reported exposure had shortened sleep duration as measured by the smartphone sensors and reported significant differences around cough and breathing with sleep suggest targets for a next phase of longitudinal and mechanistic focused research. Our lack of significant results in other domains such as mood is also notable as the personal nature of smartphone data capture enables examination of the intra-individual correlations and trajectories of illness. The challenges in characterizing PACS may be that certain aspects of the syndrome are common (e.g., sleep) while others are more variable and will require a digital phenotyping approach to find longitudinal clusters among patients.

Our results are in line with ongoing research on PACS around sleep. Active data smartphone surveys results found that sleep disturbances with individuals who perceived they were exposed to COVID-19 reported lower quality sleep and their passive data correlates supporting these results with a shorter sleep duration average of ~30 min. Similar changes in sleep have been noted in other research such as Huang et al. (19) reporting survey results of coughing and trouble breathing while sleeping, with a follow up study finding these symptoms persist after COVID-19 exposure (20). Other studies have also found self-reports of sleep have also reported greater rates of insomnia and breathing issues tied to sleep disturbances (21, 22). Our findings around sleep passive data are also in line with previous findings of PACS being associated with lower sleep quality where studies finding fatigue and sleep disturbances as prominent among PACS patients (23). Sleep related findings persist in other countries as well, which find decreased sleep quality as part of COVID-19's sequalae symptoms (24).

In this study we asked questions about prodromal psychosis with the PQ-16. There have been case reports of psychosis associated with COVID-19 and ongoing research suggests the syndrome may be associated with symptoms similar to those prodromal psychosis such as delirium and delusions (25, 26). Elevated prodromal scores may stem from stress and fear of the pandemic, which may be associated with the greater rates of self-reported psychotic symptoms. The ability of the smartphone platform to capture these symptoms may allow for new investigations on this evolving aspect of the syndrome.

In contrast to our hypothesis, students who reported exposure to COVID-19 did not spend less time at home an average than those not exposed. This could be because the public has been generally quarantining during the pandemic and because those exposed to COVID-19 may be at home as often as average as those not exposed because of subsequent quarantining after exposure. Our data and study is not designed to explore causality so we caution any further interpretation.

As proposed, our control found differences in screen time between exposure groups. While screen time has been found to be elevated during the COVID-19 pandemic due to lockdown including social isolation and greater rates of time spent at home (27), screen exposure rates may be the same regardless of condition. Other research has found that screen time has become increasingly less useful as metric of understanding mental and physical health since the rapid adoption of technology causing screen time to be high universally among populations, especially youths (28). Additionally, we did not find elevated scores in the most common mental health assessments, such as the PHQ-9 and GAD-7. These are symptoms that have often been found to be elevated during the pandemic [(4, 29)]. However, this finding is possible because the present study screened for students who were already of elevated stress levels.

Regardless, understanding COVID-19's sequalae remains a challenging and evolving question. However, smartphone-based passive data could play a key role in shedding light on this condition. Previous studies investigating passive data during COVID-19 have found similar results to our own. One study using smartwatch wearable passive data where average sleep in minutes was found to be decreased between pre- and post-lockdown (12), while another study also using smartwatch wearables found sleep differences between COVID-19 positive and negative individuals (30). While these studies support our sleep findings, our study presents a more accessible method of collecting passive data through smartphones vs. smartwatch wearables, as only 1 in 5 of Americans have found to own smartwatches or fitness trackers (31) while 85% own a smartphone (31, 32).

### Limitations and Future Directions

Our findings also present several limitations. Exposure to COVID was self-reported and not verified. Our sample was recruited from college students via the internet and thus there is a selection bias in who may have responded and agreed to partake in this work. While we studied students for ~1 month, this duration itself is likely too short to capture the trajectory of long-COVID which makes our results more exploratory in nature. Additionally, while the app in use is under consistent development and quality checks, digital phenotyping is readily evolving field and poses the question of what better methods could be used to measure the studied features.

## Conclusions

Using novel digital phenotyping methods, the present study offers supporting evidence of ongoing research regarding PACS, finding greater levels of sleep disturbances among college students. Additionally, these methods and analyses provide an example for future and larger scale studies.

## Data Availability Statement

The original contributions presented in the study are included in the article/supplementary material, further inquiries can be directed to the corresponding author/s.

## Ethics Statement

The studies involving human participants were reviewed and approved by Beth Israel Deaconess Medical Center. The patients/participants provided their written informed consent to participate in this study.

## Author Contributions

All authors listed have made a substantial, direct, and intellectual contribution to the work and approved it for publication.

## Conflict of Interest

The authors declare that the research was conducted in the absence of any commercial or financial relationships that could be construed as a potential conflict of interest.

## Publisher's Note

All claims expressed in this article are solely those of the authors and do not necessarily represent those of their affiliated organizations, or those of the publisher, the editors and the reviewers. Any product that may be evaluated in this article, or claim that may be made by its manufacturer, is not guaranteed or endorsed by the publisher.
